# Crusted and eroded plaques in metastatic lung cancer

**DOI:** 10.1016/j.jdcr.2024.10.018

**Published:** 2024-11-09

**Authors:** Robin Wang, Zisansha Zahirsha, Brandon Zelman, Jodi Speiser, Madhu Dahiya, David Eilers

**Affiliations:** a Division of Dermatology, Loyola University Medical Center, Maywood, Illinois; b Department of Pathology and Laboratory Medicine, Loyola University Medical Center, Maywood, Illinois; c Section of Pathology, Edward Hines, Jr Veterans Affairs Hospital, Maywood, Illinois; d Section of Dermatology, Edward Hines, Jr Veterans Affairs Hospital, Maywood, Illinois

**Keywords:** atezolizumab, bullous pemphigoid, immune checkpoint inhibitors, immune-related cutaneous adverse events, metastatic lung cancer, programmed death-ligand 1 inhibitor

## Case description

**Fig 1:**
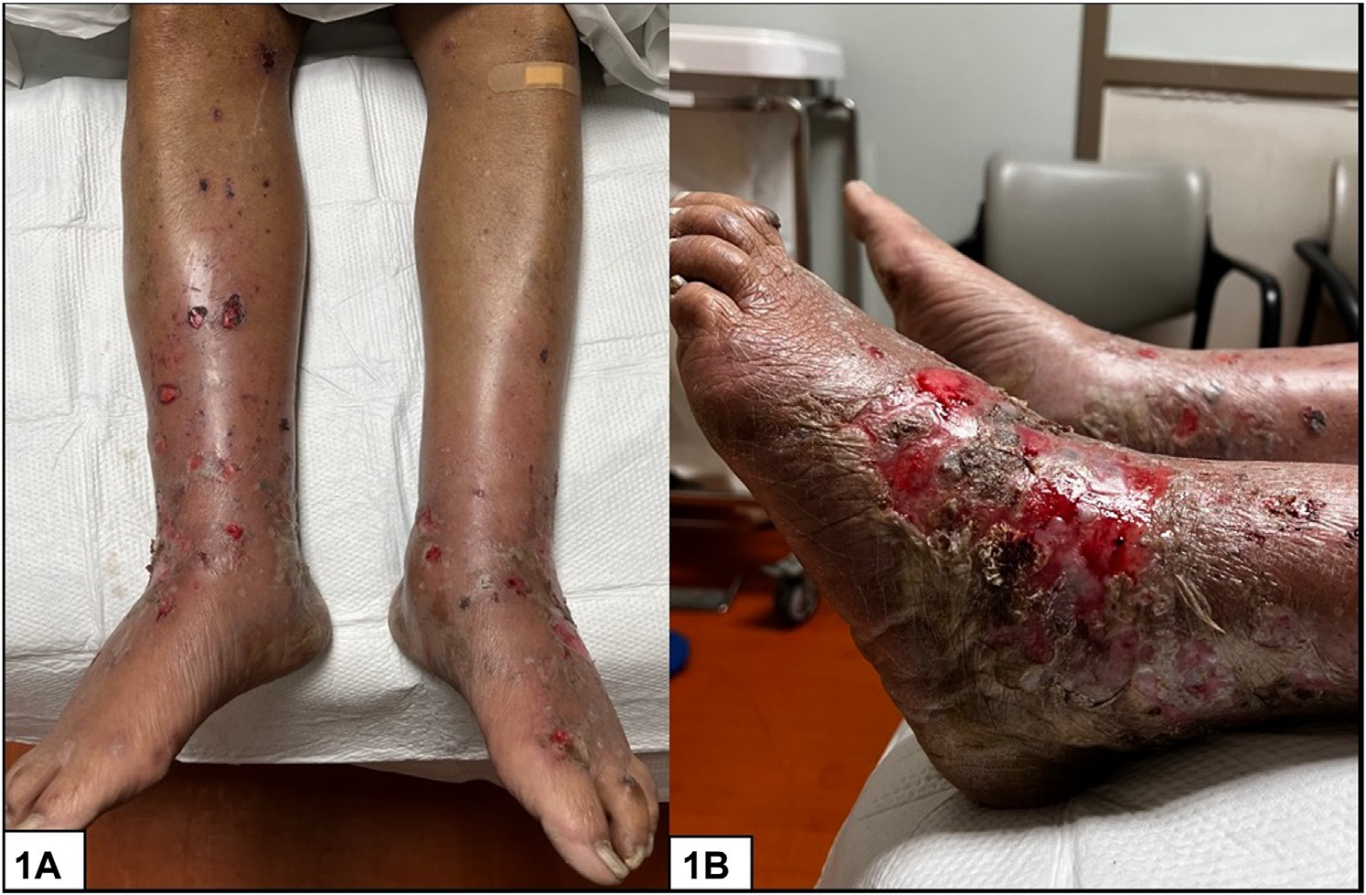

**Fig 2:**
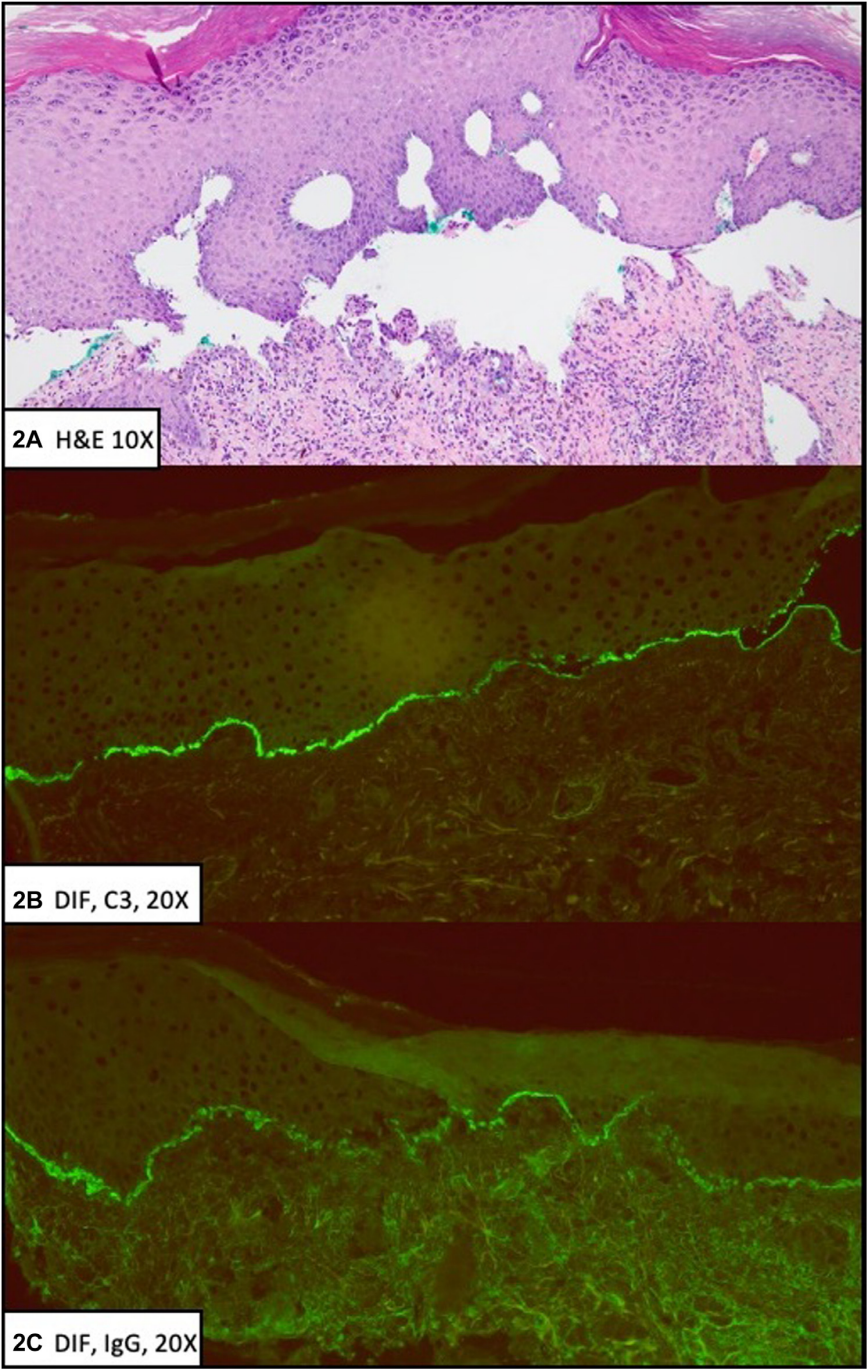

A 72-year-old man with a history of hypertension, monoclonal gammopathy of uncertain significance, and metastatic nonsmall cell lung cancer receiving atezolizumab therapy presented with a 1-week history of a painful rash on the bilateral lower extremities. The patient was admitted for presumed cellulitis and failed treatment with an empiric course of cefazolin. Cutaneous examination demonstrated multiple scattered erythematous crusted and eroded plaques on the bilateral lower extremities with confluence on the dorsal feet (Fig 1, *A-C*). Histopathology findings are demonstrated below (Fig 2, *A-C*).

**Question 1: What is the diagnosis?**

A. Stasis dermatitis
B. Erosive contact dermatitis
C. Paraneoplastic pemphigus
D. Bullous impetigo
E. Bullous pemphigoid

**Answers:**

A. Stasis dermatitis – Incorrect. Although stasis dermatitis can present with erythema, tenderness, and crusted eroded plaques of the bilateral lower extremities, the lack of edema and proximal lower extremity involvement make the diagnosis less likely. Histopathology would show spongiosis, capillary hyperplasia, hemosiderin deposition, and fibrosis of the dermis.^1^
B. Erosive contact dermatitis – Incorrect. Although erosive contact dermatitis can present with acute erythema, tenderness, and eroded plaques, the patient history and scattered distribution make the diagnosis less likely. Histopathology would show eosinophilic spongiosis with negative immunofluorescence findings.^1^
C. Paraneoplastic pemphigus – Incorrect. Although paraneoplastic pemphigus can present with polymorphous cutaneous lesions including crusted eroded plaques, early and severe mucositis is characteristic. Histopathology would show suprabasilar acantholysis, lichenoid interface dermatitis, and keratinocyte necrosis. Direct immunofluorescence would show deposition of C3 and IgG intercellularly and along the basement membrane zone.^1^
D. Bullous impetigo – Incorrect. Although bullous impetigo can present with bullae formation leading to crusted plaques, lesions are characteristically associated with a honey-colored crusting or collarettes of scale, and would respond to cefazolin therapy. Histopathology would show subcorneal bullae with neutrophils and gram-positive cocci within the blister cavity.^1^
E. Bullous pemphigoid – Correct. The patient has immune checkpoint inhibitor-induced bullous pemphigoid triggered by atezolizumab, a programmed death-ligand 1 inhibitor used for treatment of his metastatic nonsmall cell lung cancer. The patient initially developed bullae which subsequently led to the eroded plaques seen on physical exam. Histopathology demonstrates subepidermal blistering and direct immunofluorescence shows linear C3 and IgG deposition along the basement membrane.^1^

**Question 2: Which of the following is an appropriate next step in management for this patient with a grade 2 reaction?**

A. Permanently discontinue atezolizumab
B. Start triamcinolone acetonide 0.1% ointment twice daily to the affected areas
C. Temporarily discontinue atezolizumab until involvement is grade 0-1
D. Start prednisone 2 mg/kg/day plus topical high-potency steroids
E. Start rituximab

**Answers:**

A. Permanently discontinue atezolizumab – Incorrect. Permanent discontinuation of immune checkpoint inhibitor therapy is typically warranted in severe or life-threatening immune-related cutaneous adverse events (grades 3-4).^2^^,^^3^
B. Start triamcinolone acetonide 0.1% ointment twice daily to the affected areas – Incorrect. High-potency topical steroids, such as clobetasol propionate ointment, are mainstays of bullous pemphigoid treatment rather than mid-potency topical steroids. High-potency topical steroids are recommended in combination with systemic steroids for grade 2 ICI-BP reactions. The use of high-potency topical steroids alone may be sufficient to manage grade 1 ICI-BP reactions.^2^, ^3^, ^4^, ^5^
C. Temporarily discontinue atezolizumab until involvement is grade 0-1 – Correct. Grade 2 or higher ICI-BP reactions may necessitate temporary interruption of immune checkpoint inhibitor therapy until the grade of the reaction improves. In addition, treatment can include high-potency topical steroids and systemic corticosteroids such as prednisone 0.5-1.0 mg/kg/day.^2^, ^3^, ^4^, ^5^
D. Start prednisone 2 mg/kg/day plus topical high-potency steroids – Incorrect. Grade 3 and grade 4 ICI-BP reactions may necessitate higher dosages of systemic corticosteroids (ie prednisone 2 mg/kg/day), with the consideration that higher dosages have been associated with worse overall survival and interference with immunotherapy. Lower dosages of systemic corticosteroids (ie prednisone 0.5-1.0 mg/kg/day) are recommended for grade 2 ICI-BP reactions.^2^^,^^3^
E. Start rituximab – Incorrect. Due to the potential risk of serious adverse events, limited availability, and higher costs, rituximab should be reserved for grade 3 or higher ICI-BP reactions, or cases refractory to systemic steroids.^2^^,^^5^

**Question 3: Which of the following medication classes is associated with maculopapular eruption with a typical onset of 3-6 weeks after medication initiation?**

A. Aminopenicillins
B. Cephalosporins
C. Sulfonamides
D. Cytotoxic T-lymphocyte associated protein 4 inhibitors
E. Anticonvulsants

**Answers:**

A. Aminopenicillins – Incorrect. Aminopenicillins are a commonly implicated drug class in maculopapular eruptions with a typical onset of 4-14 days after medication initiation.^1^
B. Cephalosporins – Incorrect. Cephalosporins are a commonly implicated drug class in maculopapular eruptions with a typical onset of 4-14 days after medication initiation.^1^
C. Sulfonamides – Incorrect. Sulfonamides are a commonly implicated drug class in maculopapular eruptions with a typical onset of 4-14 days after medication initiation.^1^
D. Cytotoxic T-lymphocyte associated protein 4 inhibitors – Correct. Maculopapular eruption due to immune checkpoint inhibitors have an onset of 3-6 weeks after medication initiation, a later onset compared to other commonly implicated drug classes.^2^ Maculopapular eruption has been reported to affect 49% to 68% of patients receiving cytotoxic T-lymphocyte associated protein 4 inhibitor therapy and 20% of patients receiving programmed death-ligand 1 inhibitor therapy.^2^
E. Anticonvulsants – Incorrect. Anticonvulsants are a commonly implicated drug class in maculopapular eruptions with a typical onset of 4-14 days after medication initiation.^1^

## Conflicts of interest

None disclosed.

## References

[1] Bolognia J.L. (2012). Dermatology: expertconsult.

[2] Geisler A.N., Phillips G.S., Barrios D.M. (2020). Immune checkpoint inhibitor–related dermatologic adverse events. J Am Acad Dermatol.

[3] Apalla Z., Lallas A., Delli F. (2021). Management of immune checkpoint inhibitor–induced bullous pemphigoid. J Am Acad Dermatol.

[4] Merli M., Accorinti M., Romagnuolo M. (2023). Autoimmune bullous dermatoses in cancer patients treated by immunotherapy: a literature review and Italian multicentric experience. Front Med (Lausanne).

[5] Asdourian M.S., Shah N., Jacoby T.V., Reynolds K.L., Chen S.T. (2022). Association of bullous pemphigoid with immune checkpoint inhibitor therapy in patients with cancer: a systematic review. JAMA Dermatol.

